# Supplementary material for: Using touchscreen equipped operant chambers to study animal cognition. Benefits, limitations, and advice
Source: PLoS One. 2021 Feb 19;16(2):e0246446. doi: 10.1371/journal.pone.0246446 (PMC7894864; doi:10.1371/journal.pone.0246446)
Supplement: S1 File — (PDF) [file pone.0246446.s001.pdf]

S1: Detailed Grackle Training Procedure, Tips, and Tricks

Training procedures

Touch the screen to obtain food

One method for trying to get the bird to peck the digital white square on the screen was to have the experimenter demonstrate by touching the square with their finger, which would trigger the hopper, and then the experimenter could reset the hopper and show the correct behavior a few times. This drew the bird's attention to the screen, often resulting in them coming to the screen to explore it (i.e., stimulus enhancement), thus increasing their chance to learn something about the stimulus. The primary method used to encourage engagement with the screen was to put a piece of clear tape that contained bits of crushed Goldfish crackers over the digital white square on the screen. This resulted in grackles grabbing at the crackers, and coincidentally touching the screen, which triggered the hopper to raise. However, due to the sensitivity of the TOC, the tape on the screen often disrupted the "white square training" PsychoPy program because the program registered the tape as a touch and locked the hopper in a raised or lowered position. A more successful method for training the grackles to interact with the screen involved using the "hopper training" PsychoPy program, which allowed experimenters to control the hopper and it did not have a digital white square on the screen. Combined with this program, experimenters taped a paper white square onto the screen and subsequently Goldfish crackers were taped on top of the paper square. When the grackle touched the Goldfish or paper white square, the experimenter remotely controlled the hopper to make the food accessible. Once grackles were consistently pecking at the crackers taped to the paper white square, the experimenter decreased the size of the cracker crumbs until the grackle learned it would receive a reward from the hopper for simply pecking the paper white square. After the bird consistently pecked the paper white square taped over the screen and ate from the hopper, the experimenter removed the paper white square and moved to the digital "white square" PsychoPy program. If the bird did not peck the digital white square, the experimenter continued to intersperse paper white square and digital white square trials until they did.

General training tips

Although we were able to find an efficient method of training most grackles to use the TOC, throughout this discovery process there were many individual differences in the efficiency and consistency with which grackles learned to interact with the screen that required some flexibility in the training protocol. For example, some birds made pecks to the digital white square in such a way that they were not registered by the touchscreen, therefore it was important for the experimenter to remotely raise the hopper when the bird made an accurate peck to ensure the association was made between touching the white square and the food reward. Additionally, some birds pecked anywhere on the screen rather than specifically on the digital white square, so the experimenter reverted to taping food over the square to strengthen the association between the shape and the food. Some individuals took a long time to shape to the digital white square and required a significant amount of time to retrain associations before the grackle was able to consistently touch the digital white square to elicit a food reward. For

example, Habanero's later training sessions started with him pecking the paper white square taped to the screen because he didn't touch the digital white square, even though he had touched the digital white square and triggered the hopper in previous sessions. Even during later training stages, other individuals who had been comfortably interacting with the TOC sometimes reverted to fearful behavior where they would not approach the TOC during a session, or they would approach quickly and jump back rather than remaining close to interact with the stimuli on several sequential trials. In these cases, the experimenter reverted to paper white square training or hopper training until they again appeared comfortable with the TOC.

Additional training programs that were discontinued

The original idea was that it might be more ecologically relevant for the grackles to learn to interact with the TOC by learning to peck a shape that was moving around on the screen, simulating a moving insect (grackles often catch insects on the fly). Therefore, a "Moving target training" (file name: moving_stim_.psyexp) program was created and presented to two of the first grackles to be trained. Grackles were not more likely to peck a moving white circle without the extra hand-shaping training described above. Therefore, training only with the white square training program was continued because it was much easier to implement the training methods above if the stimulus did not change positions on the screen (e.g., placing paper and crackers over the location of the digital white square).

For some of the TOC experiments, the initial plan was to have a trial start key. The function of the trial start key was that a trial would only start when a bird was paying attention and ready to participate, and the bird could indicate this by pecking the trial start key to initiate each trial. When the trial start key was pecked, the test trial began. Two grackles were given (and passed) trial start key training (file name: 5. Trial_Start Key Shape.psyexp). However, as these birds moved through the experiments, the trial start key was an extra (unrewarded by food) step before the start of the trial and it negatively impacted their motivation to participate. To avoid these problems with motivation, it was deemed unnecessary to include the trial start key because the experimenter could determine when the bird was attending to the screen and trigger the trial to start from the aisle of the aviary using their computer. The trial start key was then removed from all TOC experiments. While removing the start key also removes part of the automation of this experiment, which is a large benefit of a TOC, we do not think that grackles would be able to complete experiments on TOCs in a completely automated way. This is largely due to the motivational issues we encountered, although this may be confounded by our open setup, instead of a smaller enclosure which could theoretically facilitate engagement. Thus, for the grackle experiments, the ability to remotely initiate trials was a great benefit to the TOC approach. Many researchers working with wild-caught individuals, or implementing TOC experiments in the wild will likely have similar problems. Therefore, exhaustive details of our experiences are included to showcase the flexibility of TOC methods and to facilitate other research with similar subjects.

Once grackles passed white square training, they were ready to begin participating in experiments with the TOC. However, a few additional issues were encountered. Originally, the reversal learning TOC experiment included two different colors and required color discrimination (see Logan et al. 2019a). However, the first two birds that passed TOC training and moved on to the reversal TOC experiment exhibited avoidance and/or fearful behaviors when presented with a light purple circle and a dark purple circle simultaneously on the screen. They refused to come back to the TOC area even after attempts to habituate them to a variety of colors and shapes on the screen. Therefore, the reversal TOC experiment was changed to instead discriminate between two different white shapes.

S2. TOC Training Protocol for Experimenters to Follow

Developed by: CJ Logan, KB McCune, M MacPherson, L Bergeron, B Seitz, A Blaisdell
The Grackle Project, www.CorinaLogan.com, January 2019, updated November 2019

Touchscreen set up: The touchscreen apparatus is mounted on a platform (46 cm wide x 70 cm long x 8 cm tall) placed on a cart (89 cm tall) inside an individual subject's aviary. All stimuli are presented by computer on a color LCD monitor (NEC MultiSync LCD1550M). Pecks to the monitor are detected by an infrared touchscreen (Carroll Touch, Elotouch Systems, Fremont, CA) mounted in front of the monitor. A food hopper (Coulbourn Instruments, Allentown, PA) is located below the monitor with an access hole situated flush with the floor. When in the raised position, the hopper provides access to pieces of Goldfish crackers. All experimental events are controlled and data recorded with a laptop (Dell Inspiron 15). All experimental procedures are programmed using PsychoPy v.1.85.2 (http://psychopy.org; our programs only work on this version of PsychoPy). The touchscreen is remotely controlled using TeamViewer.

Files (.psyexp) located at: Grackle2 (a laptop) > Desktop > Touchscreen Experiments > Touch screen training (note: use the Grackle2 computer, not Grackle1)

Mirror displays: The apparatus (touchscreen & Grackle2 laptop) should mirror displays automatically if everything is plugged in to the outlet (has power) and to the laptop prior to turning on the laptop. Otherwise, you will have to navigate on the laptop trackpad while looking at the connected touchscreen monitor: right click, scroll to Display Settings and select 'Mirror Displays' and then click to keep the changes.

Video recording: Only video record sessions where you are trying to get the bird to pass criterion. Once they pass, progress to the next training program or test. NOTE: if a bird passed criterion the previous day, re-run that program to make sure they retained the information before moving on to the next program.

Enter data at: data_TouchScreenTraining

TeamViewer

Experimenters need to **download** TeamViewer (https://www.teamviewer.com/en-us/, the version for personal use) to their computer (not to their smartphone because it doesn't work with our experiments) to be able to remotely control the touch screen. Get the touch screen **computer ID and password** by opening TeamViewer on the touch screen computer. Make sure to **mute** sounds in TeamViewer on your remote computer.

Prep the aviary

Prior to beginning a session, remove maintenance diet from the aviary and record the time in the data sheet. Many individuals will be more willing to work when they have been deprived of food for 1+ hours, and the maximum time an individual can be deprived of maintenance diet is 4 hours. Sweep up any spilled maintenance food from the floor so the individual only has access to food from the touch screen.

Training: food hopper

Habituation to the food hopper: It makes training go faster if they are fed from the food hopper overnight before they begin hopper training (Figure 1). It can take multiple days before they are habituated to the whole touch screen apparatus. Record habituation in data_TouchScreenTraining (TrainingType=Habituation).

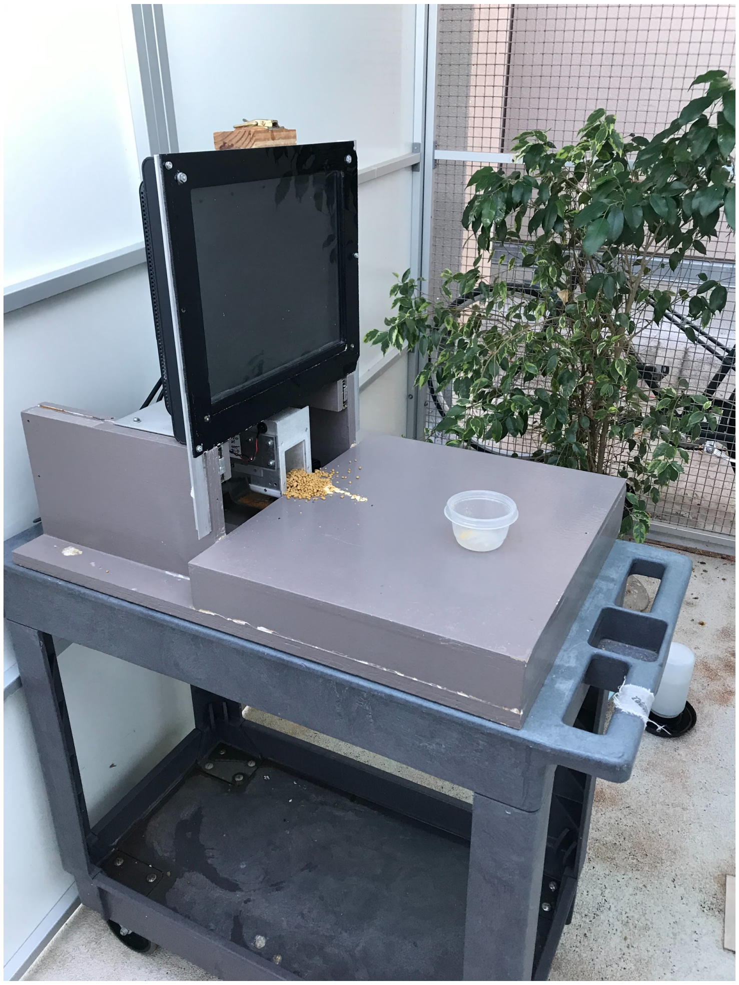

Figure S1. Habituation to the touch screen apparatus and to eating from the food hopper. All food is in the hopper area and the maintenance diet food dish is empty so that they can only eat in or near the hopper. Note: Face the touchscreen the appropriate direction for each individual following Table 1 in the Causal Cognition protocol.

Summary: The aim is to get the grackles to associate the sound of the hopper moving with food being available (note: a light also turns on when the food hopper is available, however this experiment is conducted in outdoor aviaries where it is bright and thus the light might not be the most obvious cue). Every time the grackle approached the food hopper (e.g., landed on the platform, progressing to when they approach the hopper), we remotely moved the hopper into the available position so the bird could eat. The movement of the hopper makes a distinct sound. The hopper is kept in the available position until the grackle looked inside, and then the hopper was made unavailable. End goal behavior for hopper training: grackle lands on platform, hopper is made available, grackle eats food from hopper, hopper is moved out of reach, grackle is unable to obtain food.

Table S1. Components that comprise the apparatus and instructions on how to put them together.

**Component**		
Touchscreen with hopper		
Grackle2 laptop (with power cable)		
Cord 1 (power to hopper)	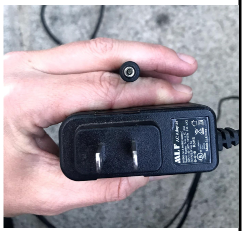	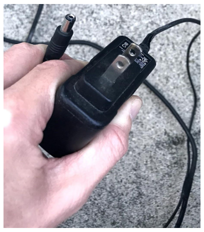
Cord 2 (hopper to laptop)	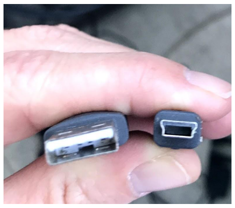	
Cord 3 (laser screen to laptop)	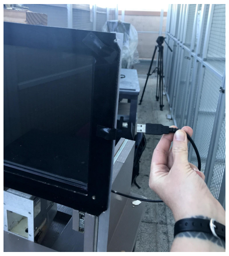	
Cord 4 (monitor to laptop - x cord & adapter)	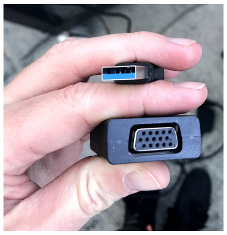	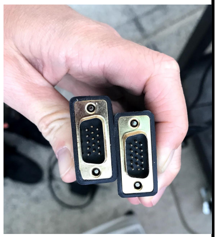
Cord 5 (power to monitor)	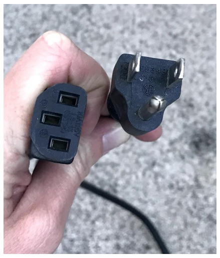	

A second laptop with TeamViewer installed		
An extension cord with at least 3 plug-ins for the 3 power cables needed for this apparatus		

A 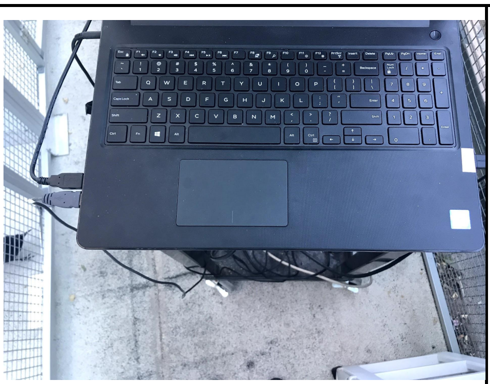	B 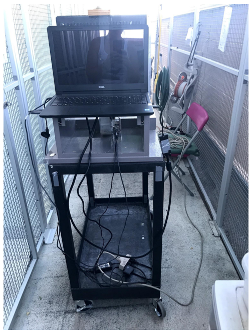	C 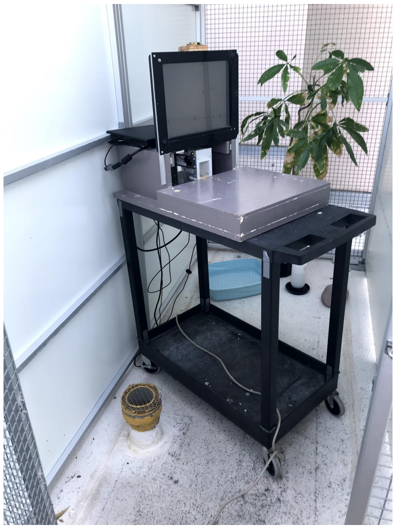
D 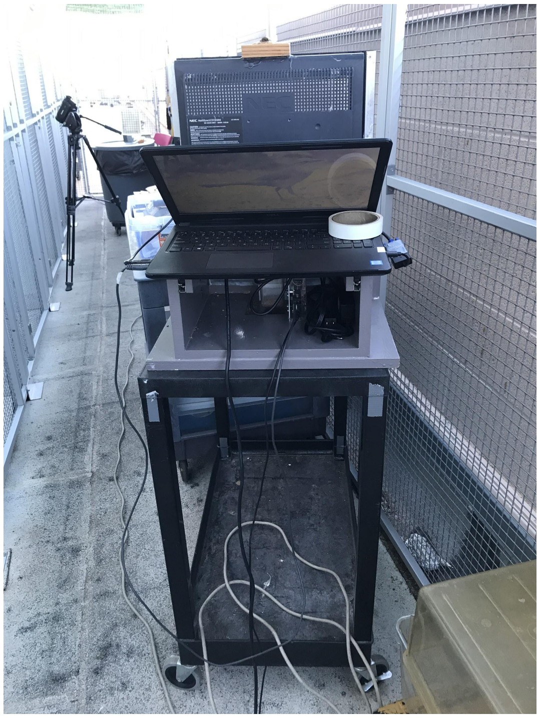	E 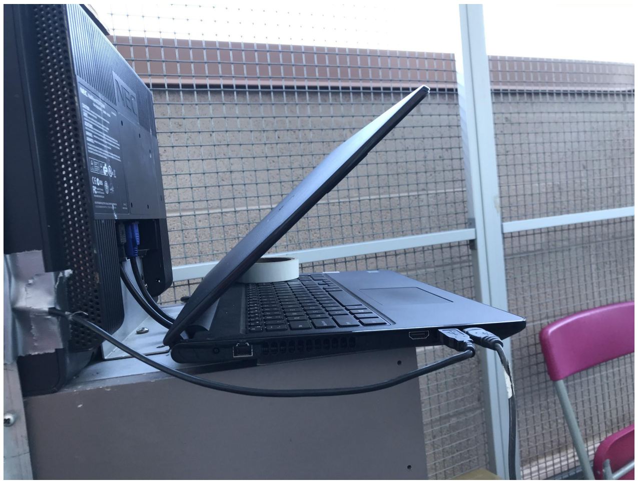	

Figure S2. **A)** A visual of what the touch screen laptop looks like with everything plugged in. The specific cables need to be plugged into specific com ports otherwise changes need to be made to the PsychoPy code (clockwise from bottom left, the items plugged into the USB ports are: food hopper (left, closest to operator), touchscreen (left closest to touchscreen), monitor (right only USB port)). **B)** A visual of what the computer and cords look like when all are connected. The end of the extension cord is placed on the bottom shelf of the cart when wheeling the cart into the aviary, as are the cords connected to it so that only a single cord breaches the aviary doorway when the cart is moved into the aviaries for testing. **C)** The set up inside the aviary. The cart is pushed at an angle to the left wall and the laptop is left open by being propped up against the wall's middle column. The cords are tucked up underneath the bottom shelf to avoid distracting the birds during training and testing. **D and E)** A roll of masking tape is used to prevent the laptop screen from closing. If the screen closes, the stimuli on the screen are in the wrong places.

Hopper Habituation Training

Do not video record these sessions.

NOTE: when using the touchscreen, do not close the Dell laptop lid! Doing so will make all of the stimuli off center in programs where digital stimuli are presented.

Record data for steps 1-3 on a single line per session with details listed in the notes column in data_TouchScreenTraining.

- PsychoPyFile = 1.Press_Space_for_food_2.Basic_mag_training_nostartscreen.psyexp (Note: previous to 8 Jan 2020 this program was used: 1.Press_Space_for_food_2.Basic_mag_training_.psyexp)
- TrainingType = Hopper Training
- Each session should be noted in "Session", along with its start and end times.
- Note the first trial of each session in "Trial", but each trial's details should be listed in the notes column.
- Keep track of the number of days it takes a bird to habituate to the hopper, the amount of time per session and roughly the number of trials attempted (by both the experimenter and the participant).
- Do not record data in the Choice column

The training

1. Outside of the aviary: On the Dell laptop, start the program **1.Press_Space_for_food_2.Basic_mag_training_.psyexp** (https://github.com/corinalogan/grackles/blob/master/Files/TouchscreenPsychoPy2code/TouchScreenTraining/MovingTargetTraining/maestro.py). To start the program, choose it from the folder: TouchScreen programs (on Grackle2's desktop). Session name = leave blank. The timing of the hopper movements is automatically recorded by PsychoPy, which generates an Excel file in the folder TouchScreen programs > Data. An Excel file is made every time a PsychoPy program is run. When we are not attempting to get a bird to pass criterion, delete these excel files to avoid later confusion.
2. Position the food hopper so it is in the accessible (raised) position from the beginning of the session.
    a. Bring the touchscreen in to the aviary and face it in the appropriate direction for each individual (see Table 1 in the Causal Cognition protocol).

   b. Draw attention to the hopper at first by placing food crumbs around the area. Allow the bird to eat from the hopper for 20 seconds, then go into aviary and add more crumbs at/around hopper. Repeat until the bird eats from the hopper without the crumbs. Note: This first step does not entail lowering or raising the hopper. It is simply about attracting the individual to the hopper for food.
3. Once the bird is comfortably eating from the hopper, habituate the bird to the sound of the hopper moving. Use the **1.Press_Space_for_food_2.Basic_mag_training_.psyexp** program which lets you press the spacebar to raise, and lower the hopper. Raise and lower the hopper many times when the bird is attending to the apparatus, especially when the bird is on the platform.
   a. Allow the bird to come to the platform and eat from the raised hopper (as in the previous step). Immediately after it eats, press the spacebar to lower the hopper while the bird is watching. Repeat until the bird is no longer jumpy. In this first step, the hopper is already raised when the bird comes to the platform.
   b. Next, with the hopper set to the lowered position, raise the hopper after the bird lands on the platform so that the sound of the hopper being raised cues them that food is becoming available. This helps train the bird to 1) go to the hopper to look for food when they hear the sound of the hopper moving, and 2) that food is not always available.
4. To train the grackle to eat quickly from the hopper, the experimenter continues to use the **1.Press_Space_for_food_2.Basic_mag_training_.psyexp program**. Again, use the spacebar to raise the hopper into the accessible position when the grackle is on the platform, allow the hopper to be available for 20 s, then move the hopper out of reach. Wait 5-10 seconds (so the grackle has time to notice food is no longer available), then initiate another trial by making the hopper accessible again. Allow the grackle to eat for 5-20 s (or until it is seen with 3 food items in its bill, so it has eaten at least 3 food items). Repeat. At first, let the food stay available for 20s and gradually decrease it to ~8 s (which is what it will be during testing). Gradually decrease this food availability period until the grackle does not retreat or show signs of fear (e.g., flying away, jumping backwards, reluctant to return to hopper, reluctant to put head in hopper). If grackle leaves the platform, make the food unavailable and only make it available again when the grackle is on the platform facing the hopper.
   a. Once the grackle has habituated to going to and eating from the food hopper when it is raised, proceed with trials to assess whether grackle passes hopper training

Hopper training passing criterion: 17/20 trials correctly getting food from the hopper

Video record all sessions
Record data at data_TouchScreenTraining

- one row per trial
- start with session 1, trial 1
- TrainingType = Hopper Criterion

Record Choice as follows:

1. 1 = the grackle ate from the hopper within 5 s of the food becoming available
2. 0 = the grackle did NOT eat from the hopper within 5 s of the food becoming available
3. -1 = the grackle left the platform after the hopper was raised or otherwise did not make a choice; repeat the trial the next time the grackle comes to the platform

Back up the data: save the data sheets generated by PsychoPy (on Grackle2's Desktop in Touchscreen Experiments > Touch screen Training > data) to Keeper and to an external hard drive. Rename these files in the following format: A073OL hopper training S1 T1_2019_Nov_07_1445.csv.

Video File naming convention: A073OL 2019-11-06 hopper training S1 T1

Psychopy excel output file naming convention: A064LR 2019-11-25 hopper training S1 T1
PsychoPy automatically saves the file using the naming convention entered when opening the program, then it adds the date and time the program ran.

Conducting a session

1. Outside of the aviary on the Grackle2 laptop: Run the PsychoPy hopper training program: **1.Press_Space_for_food_2.Basic_mag_training_.psyexp program**. Participant = bird ID and session and trial number (e.g., A073OL hopper training S1 T1 indicates the first session and first trial of hopper training trials). Once the program is running, turn on the camera and use the white board to indicate date, bird ID, experiment (Hopper Training), session number, trial number and experimenter.
2. Bring the touchscreen into the aviary and face it in the appropriate direction for each individual (see Table 1, Causal Cognition protocol). Put the touch screen at an angle in the aviary so that the camera can see the screen and also the position of the food hopper (e.g., the camera should be higher than the touchscreen platform). If needed, put a small cracker piece in front of the hopper before leaving the aviary to entice the bird to quickly begin trials.
    a. Once the grackle comes to the platform, let it take the free piece of cracker, then press the spacebar to raise the hopper.
    b. Count to 5. If the grackle sticks it's head in the hopper, let it eat <u>up to</u> 3 pieces of food before lowering the hopper.
    c. Lower the hopper and count to 10 before raising the hopper again for the next trial.
    d. If the grackle does NOT stick its head into the hopper within 5 seconds, lower the hopper. Count to 10 before raising the hopper again for the next trial.
        i. End the session and try again after giving the grackle a break if the they left the platform and did not come back within 5 min of the start of the previous trial.
3. ***Criterion:*** Subject needs at least 17 of the most recent 20 trials correct (obtaining the food with the hopper moving forward and backward at maximum speed), with at least 8/10 or 9/10 correct in the most recent two 10 trial blocks (as in Bateson et al. 2015 PLOS ONE). Intertrial interval = 10 s (food is not available during this time so the grackle learns it must pay attention to when it is available).
    a. NOTE: if a bird passed criterion the previous day, re-run this program to make sure they retained the information before moving on to the next program.
    b. The timing of the 5 seconds of hopper availability per trial and the 10 second inter-trial interval is counted by the experimenter silently or using a stopwatch.

How to adjust the hopper height

IF A BIRD CAN ACCESS THE HOPPER WHEN IT IS LOWERED, THIS MUST BE FIXED so they can learn that touching the screen is the action that results in a food reward. Below are instructions for how to do this in Psychopy.

Within the element code_4:

```
if FeederClosing.status == STARTED:
   servo.setAccel(0,-2)      #set servo 0 acceleration to -2
```

```
servo.setTarget(0,6000)  #move servo to resting position
servo.setAccel(10,-2)      #set servo 0 acceleration to -4
servo.setTarget(10,4500)
servo.close
```

- Change the 6000 value to something larger like 8000 to lower the resting position of the hopper. You might have to play around with this value, but larger means further away from the hole.

Additionally, adjust the 6000 to 8000 in the beginning of the experiment on the routine called slide_two_move_servo2rest.

Within the element slide_two_move_servo2rest

```
import maestro
servo = maestro.Controller('COM6')
servo.setAccel(0,-2)      #set servo 0 acceleration to -4
servo.setTarget(0,6000)
servo.setAccel(10,-2) #for the light
servo.setTarget(10,4500) #turn the light off
```

- Again, adjust the 6000 value to 8000 or some other number that sends the hopper further from the hole.
- Note: The value of 8000 brings the hopper all the way down to the base of the platform it sits on (i.e., the plastic container with food pieces touches the wooden base) AND pulls it a little further back away from where the birds access the hopper. It may be possible to use an even higher number than 8000 to pull the hopper even further out of reach if some individuals can still reach the food.

Training: touch screen

General Notes

- Once a bird is habituated to the food hopper and touchscreen apparatus and has passed hopper training criterion, **only put the touchscreen in their aviary when you want them to pay attention to it and, if they make the correct response, they get a reward**. Because hand shaping works best for training grackles on the touchscreen, all training sessions must be attended by the experimenter who must pay attention the entire time (see the video on "how Dazzle learns to blow bubbles" to learn about how hand shaping works: http://www.dogtrainingology.com/concepts/shaping-behavior-definition/).
- If a bird is having trouble with motivation/focus:
  - Take them back to the last program they were successful at and let them have a few good trials before trying the more advanced program. Also, if they are giving up on participating in a program because they are frustrated, end on a good note by taking them back to a program they already know and give them a few successful trials to make sure they stay interested in interacting with the touchscreen
  - Demonstrate yourself how to use the program by touching the screen when the grackle is watching
  - If the bird touches the correct stimulus on the screen, but the touch does not activate the hopper due to a program error, use your cursor to click the button for them so they are rewarded for the correct behaviors and continue to progress with their learning.

Goal behavior and how to start training: For touchscreen training, the bird will have to peck a digital white square on the touchscreen, which will trigger the hopper to make food available so the grackle can receive a food reward. There are a few ways that one can proceed to get the bird to peck a white square on the screen. One option is to start with the instructions for "Paper white square…". Another option is to start with the instructions for "Digital white square...". The choice depends on the experimenter's preferences and how the bird is to work with.

NOTE: when using the touchscreen, do not close the Dell laptop lid! Doing so will make all of the stimuli off center in programs where digital stimuli are presented.

Paper white square: Tape white paper to the screen while using the hopper training program to train grackles to touch the screen

Do not video record sessions.
Record data in data_TouchScreenTraining (one row per session, trial notes in Notes)

- TrainingType = Paper Square

PsychoPy program: 1.Press_Space_for_food_2.Basic_mag_training_.psyexp

Summary: tape a white paper square to the screen directly over where the digital white square would be (in 4.Food_Key_Only_2FullControl.psyexp). Place a tape hammock onto the paper white square, and put a piece of Goldfish cracker inside the tape hammock (hammock instructions below). Every time the participant touches the cracker or anywhere on the white paper square, the experimenter presses the spacebar to raise the hopper. This trains the bird to peck the white paper square to raise hopper to make it easier to transfer them to pecking a digital white square (as in the 4.Food_Key_Only_2FullControl.psyexp program). Once the bird consistently pecks the white paper square without a baited tape hammock, they progress to the digital white square program.

Note: When training multiple birds at a time on hopper or touchscreen training, the white paper square can easily be removed and reapplied while the same 1.Press_Space_for_food_2.Basic_mag_training_.psyexp program is running. For improved efficiency, leave the program running while the touchscreen apparatus is moved between aviaries.

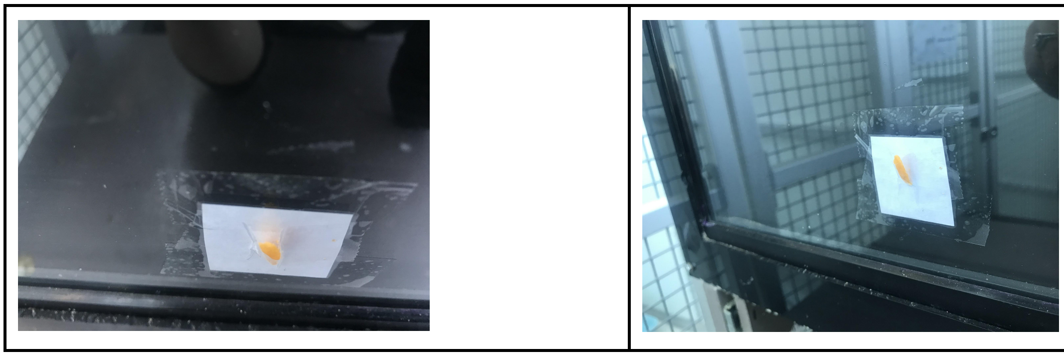

Figure S3. The white paper square with a tape hammock containing a piece of Goldfish cracker. This is useful for hand shaping participants to pay attention to the touchscreen.

Digital white square: Use the *peck food key for food* program to train grackles to touch the screen

Video record sessions when attempting to meet criterion.
Record data in data_TouchScreenTraining

- TrainingType = Digital Square - OR - Tape Hammock (depending on what was used in a given trial)
    - The experimenter can switch between these two training types within a session and without giving the bird a break between sessions, just make sure to make a new row for this session when switching between trial types.
- TrainingType = White Square Criterion (when attempting to get the bird to pass criterion)

PsychoPy program: 4.Food_Key_Only_2FullControl.psyexp
Video file naming convention: A073OL 2019-11-07 Touchscreen Training White Square S1 T1

Summary: A digital white square (3.5 cm by 3.5 cm) is on screen and, when pecked, it disappears and the food hopper automatically becomes available. The experimenter must press the spacebar to make the food unavailable, and then the digital white square re-appears. Experimenter can raise or lower the food hopper at any time by pressing space bar.

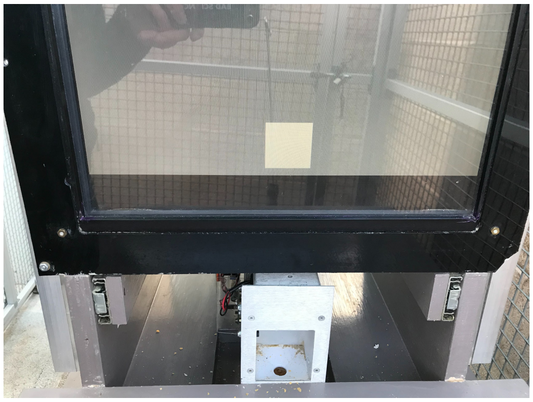

Figure S4. Digital white square food key.

Conducting a session

- Begin the program from outside of the aviary as above. Face the touchscreen the appropriate direction for each individual (see Table 1, Causal Cognition protocol). To get the grackle interested in the screen, tape a goldfish cracker to the screen on top of the white square.
    - Try to create a **tape "hammock"** so that the sides and bottom are taped on, but there is a gap in the center top where you can drop in crackers
    - Put a cracker piece in the tape "hammock". When the grackle takes the piece, press the spacebar to raise the hopper
        - Let the grackle eat 2-3 pieces from the hopper before pressing the spacebar to lower it
        - If the grackle does not notice the hopper, lower it after about 8 s
        - If they don't come back to the table, bait the tape again with a very small cracker
    - Once the grackle is taking the cracker comfortably, reduce cracker size to just crumbs
    - Once the grackle is picking at the tape piece consistently, halve the size of the tape piece
        - Continue decreasing the size of the tape piece until they peck the digital square with no tape on the screen. If they lose motivation, revert to putting cracker crumbs in the tape piece.
    - Another way to get the grackle to associate the digital white square with food is to hand shape by rewarding (giving them enough time to eat a couple of pieces of food) when the bird's bill is near the white square, and then when it is closer to the square, and then when it is touching the square. The hopper automatically comes up when the bird pecks the square, but if it doesn't and the bird gave the correct response, then press the spacebar to move the hopper up. The experimenter will need to press the spacebar to move the hopper out of reach after the bird gets one or two pieces of food.

4. ***Criterion:*** Subject needs at least 17 of the most recent 20 trials correct (with the hopper moving up and down at maximum speed), with at least 8/10 or 9/10 correct in the most recent two 10 trial

blocks (as in Bateson et al. 2015 PLOS ONE). Intertrial interval = 10 s (food is not available during this time so the grackle learns it must pay attention to when it is available).

a. All trials that count toward criterion must not have any tape or bait

Bird is now ready for the go no-go touchscreen experiment.

Training: peck trial start key to initiate white square - we NO LONGER do this training

We originally thought that the touchscreen experiments would be more automated and so we had this training program in the protocol for the bird to indicate when they were attending to the screen. However, the experimenter now manually initiates all trials from the aisle of the aviaries when the bird is attending to the screen. Therefore, we do not use this training program anymore (it was disused after April 2019).

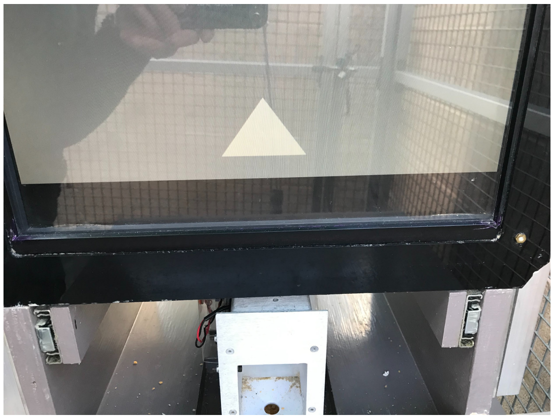

Figure S5. White triangle start key.

Program: 5. Trial_Start Key Shape
(https://github.com/corinalogan/grackles/blob/master/Touchscreen_PsychoPy2_code/TouchScreenTraining/Shaping/4.%20Start%20Key%20Shape.psyexp)

NOTE: when using the touchscreen, do not close the Dell laptop lid! Doing so will make all of the stimuli off center in programs where digital stimuli are presented.

How it works: A white triangle (5 cm base, 3 cm height) flashes for 5 s, stops for 5 s if not pecked, then flashes again. If peck is made, bird has 20 s to peck food key (3.5 cm by 3.5 cm), which should automatically trigger the food hopper (hopper available for 10 s. To make it longer, change the duration of the FeederOpening text and key_resp_2 element to the desired duration). If the food hopper does not automatically activate, the experimenter can open and close the hopper manually using the spacebar.

a. The purpose of this training is for the bird to learn to peck the trial start key (white triangle) which, during testing, will not result in a food reward, but will result in the beginning of a trial. This program starts with a flashing white triangle (on for 0.7s, off for 0.3s as in Bateson & Kacelnik, 1995) centered near the bottom of the screen. Once pecked, the white triangle disappears and a white square appears in the triangle's location and in a fixed location that will be utilized in additional experiments. The correct response is for the bird to peck the white square, which results in receipt of a food reward.

b. ***Criterion:*** Subject needs at least 17 of the most recent 20 trials correct (with the hopper moving forward and backward at maximum speed), with at least 8/10 or 9/10 correct in the most recent two 10 trial blocks (as in Bateson et al. 2015 PLOS ONE). Intertrial interval = 10 s (food is not available during this time so the grackle learns it must pay attention to when it is available).

c. Once criterion is met, begin the causal cognition test. NOTE: if a bird passed criterion the previous day, re-run that program to make sure they retained the information before moving on to the next program.

d. If a bird has used this program proficiently but then appears to not remember how to use it, run a few trials with the 4.Food_Key_Only_2FullControl program to refresh their memory and/or re-engage their interest, and then go back to this program immediately afterward.

S3. PsychoPy coding tips

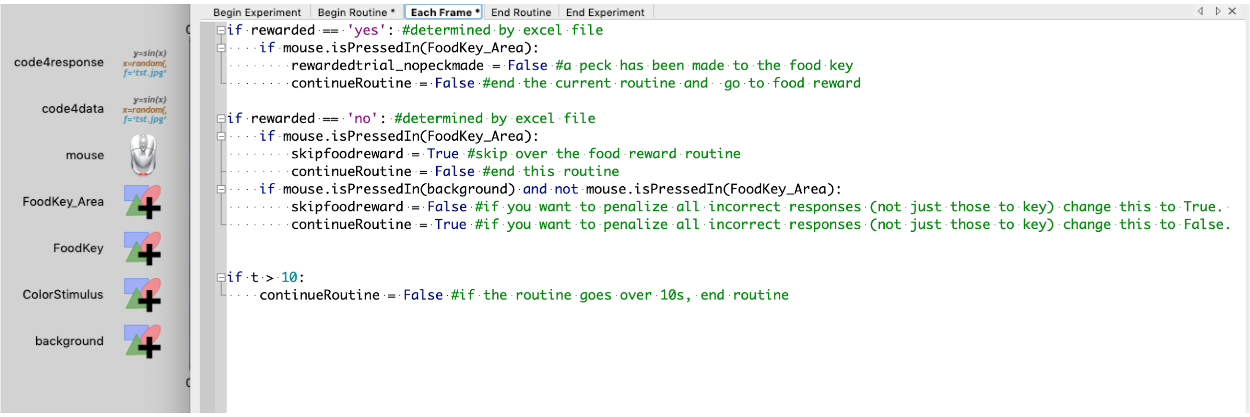

S6: Screenshot of Go/No-Go code in PsychoPy. This code governs the Go/No-go trial and determines whether the subsequent foodreward routine should continue or be skipped over.

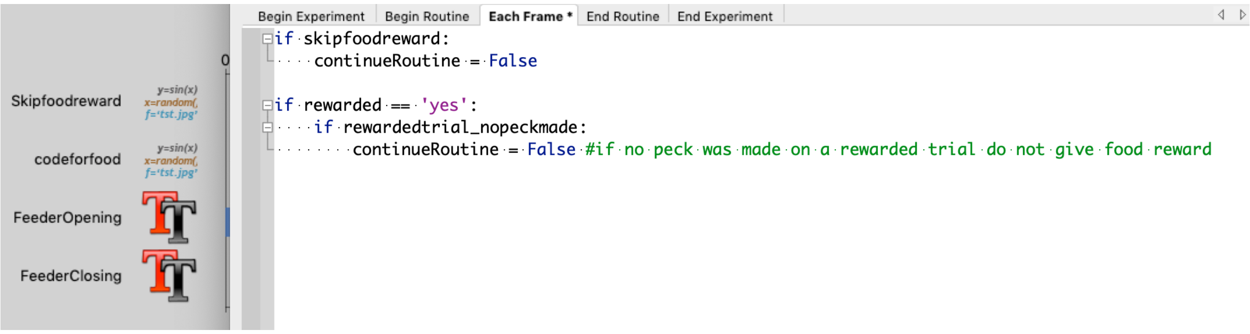

S7: Screenshot of the food reward code in PsychoPy. If the cause skipfoodreward has been made true, the Rroutine immediately ends and no food is delivered.

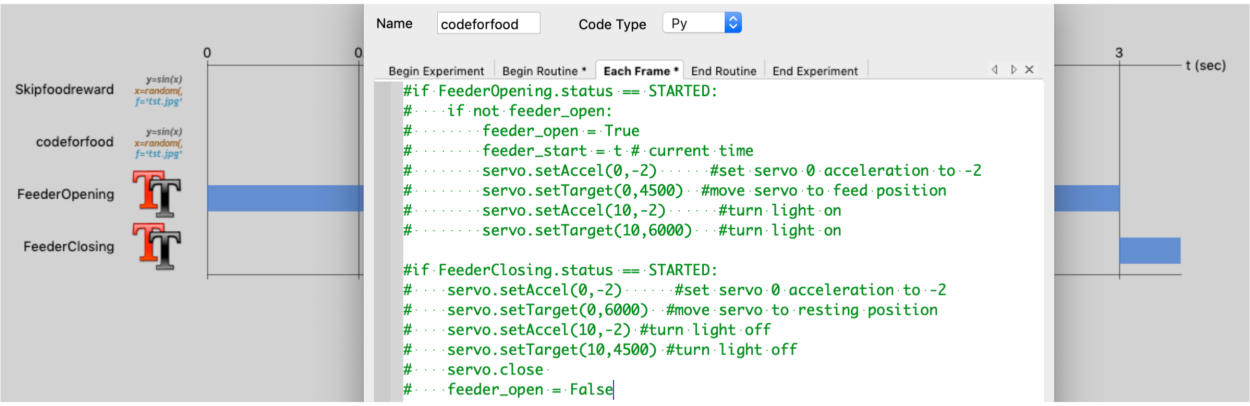

S8: Screenshot of code to move the robotic food hopper (also called Servo) in PsychoPy. By making the hopper movements contingent on more simple elements like a textbox, we were able to test the program on computers not hooked up to the actual TOC. To increase the duration of the food reward, we could simply adjust the duration of the FeederOpening text element as well as the start time of the FeederClosing text element.
